# Supplementary material for: Quantitative effects of environmental variation on stomatal anatomy and gas exchange in a grass model
Source: Quant Plant Biol. 2022 Mar 9;3:e6. doi: 10.1017/qpb.2021.19 (PMC10095872; doi:10.1017/qpb.2021.19)
Supplement: Supplementary file 1 [file S2632882821000199sup001.zip › S2632882821000199sup002.docx]

**Supplementary figures**

**
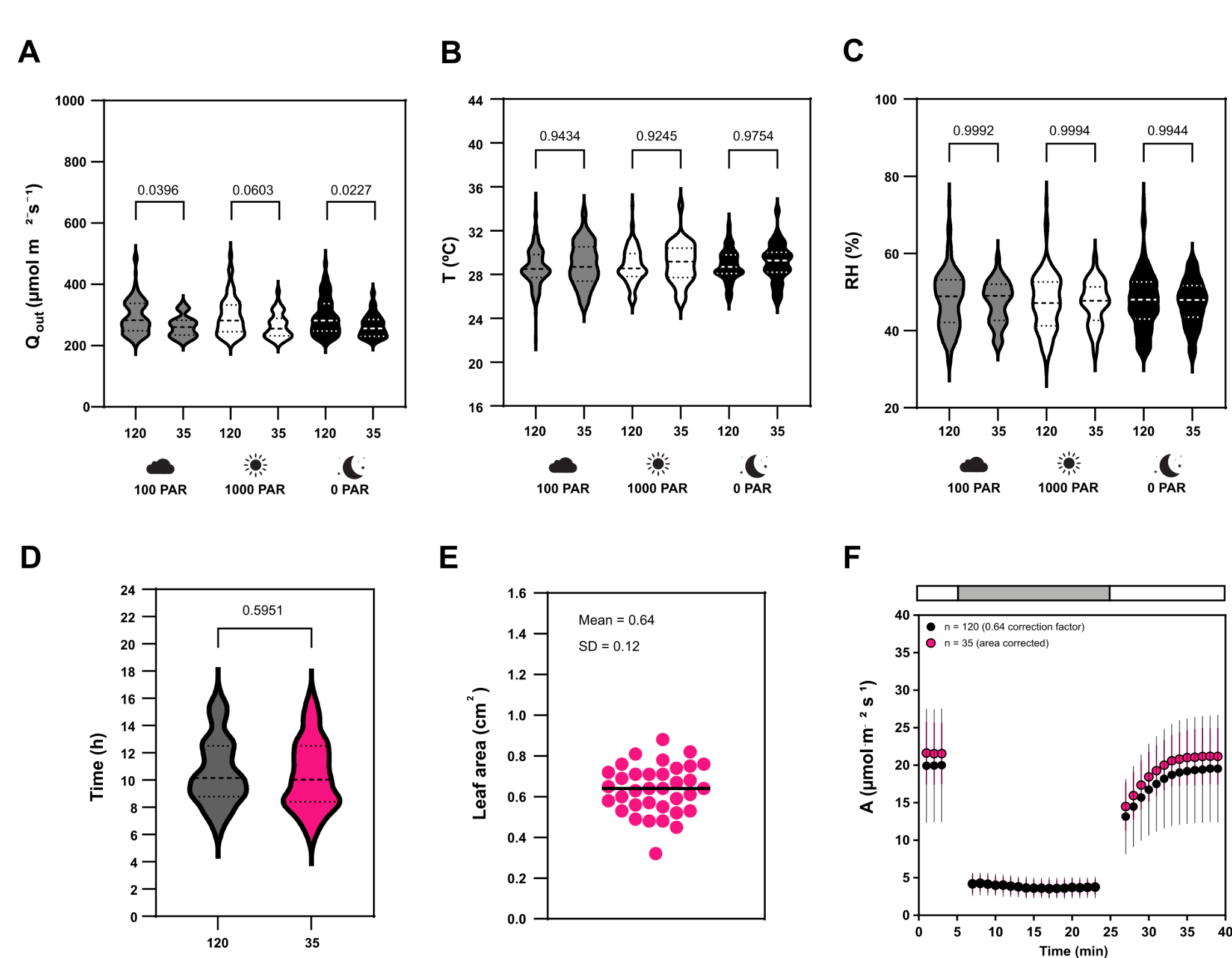
**

**Figure S1 - Environmental conditions in the greenhouse, leaf area and carbon assimilation.** (A) Ambient light during LI-6800 measurements (last 5 min of each steady-state used for correlation analysis at 100, 1000 and 0 PAR; both the total set of 120 measurements and the area-corrected subset of 35 individuals are shown). (B) Temperature during LI-6800 measurements (last 5 min of each steady-state used for correlation analysis 100, 1000 and 0 PAR; both the total set of 120 measurements and the area-corrected subset of 35 individuals are shown). (C) Relative humidity during LI-6800 measurements (last 5 min of each steady-state used for correlation analysis 100, 1000 and 0 PAR; both the total set of 120 measurements and the area-corrected subset of 35 individuals are shown). (D) Range of starting time of Li-6800 measurements of the 120 individuals (grey) and of the data subset of 35 individuals (magenta). (E) Leaf area accurately measured in a subset of 35 individuals (0.64±0.12). (F) Carbon assimilation during the light transitions 1000-100-1000 PAR (in black, data from 120 corrected by average leaf area of 0.64 cm^2^ and in magenta data from a subset of 35 individuals corrected by individual leaf area). P-values from t-tests are indicated.

**
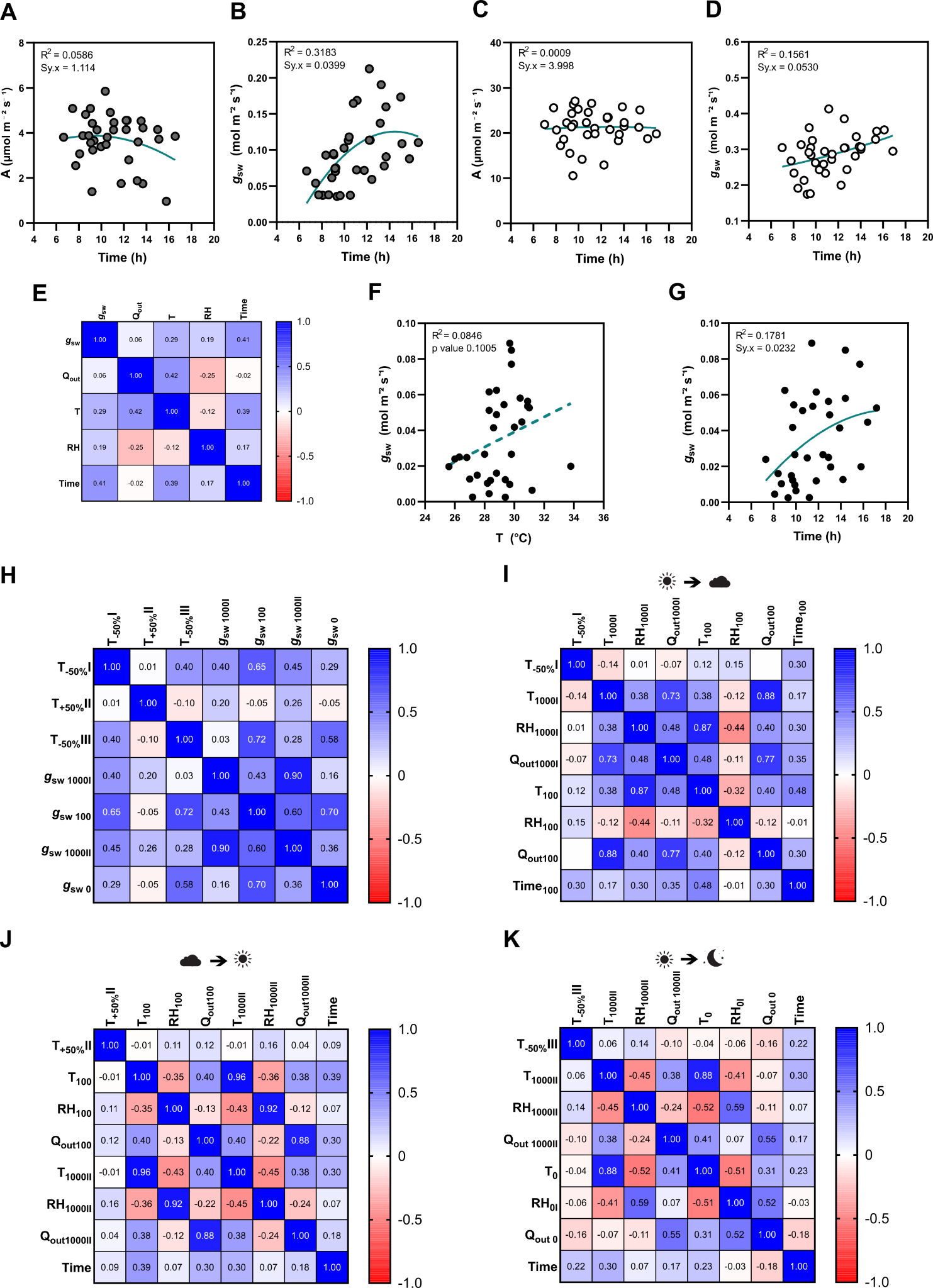
**

**Figure S2 - Effect of environmental conditions on gas exchange and stomatal kinetics.** (A) Non-linear relation between *A* and time at 100 PAR (n=35). (B) Non-linear relation between *g*_sw_ and time at 100 PAR (n=35). (C) Non-linear relation between *A* and time at 1000 PAR (n=35). (D) Non-linear relation between *g*_sw_ and time at 1000 PAR (n=35). (E) Correlation matrix between *g*_sw_ at 0 PAR and environmental conditions (Q_out_, T, RH and time) (n=35). (F) Linear relation between *g*_sw_ and Q_out_ at 0 PAR (n=33). (G) Non-linear relation between *g*_sw_ and time at 0 PAR (n=33). (H) Correlation matrix between stomatal half-time of the light transitions 1000-100 PAR(T_-50%_I), 100-1000 PAR (T_+50%_II) and 1000-0 PAR (T_-50%_III) and initial/final step steady-state *g*_sw_ (n=35). (I) Correlation matrix of stomatal closure half-time during the first light transition from 1000 to 100 PAR (T_-50%_I), T (1000 and 100 PAR), RH (1000 and 100 PAR), Q_out_(1000 and 100 PAR) and time (n=120). (J) Correlation matrix of stomatal opening half-time during the second light transition from 100 to 1000 PAR (T_+50%_II), T (100 and 1000 PAR), RH (100 and 1000 PAR), Q_out_(100 and 1000 PAR) and time (n=120). (K) Correlation matrix of stomatal closure half-time during the third light transition from 1000 to 0 PAR (T_+50%_II), T (1000 and 0 PAR), RH (1000 and 0 PAR), Q_out_(1000 and 0 PAR), time (n=120). R^2^, and Sy.x or p-values are indicated.

**
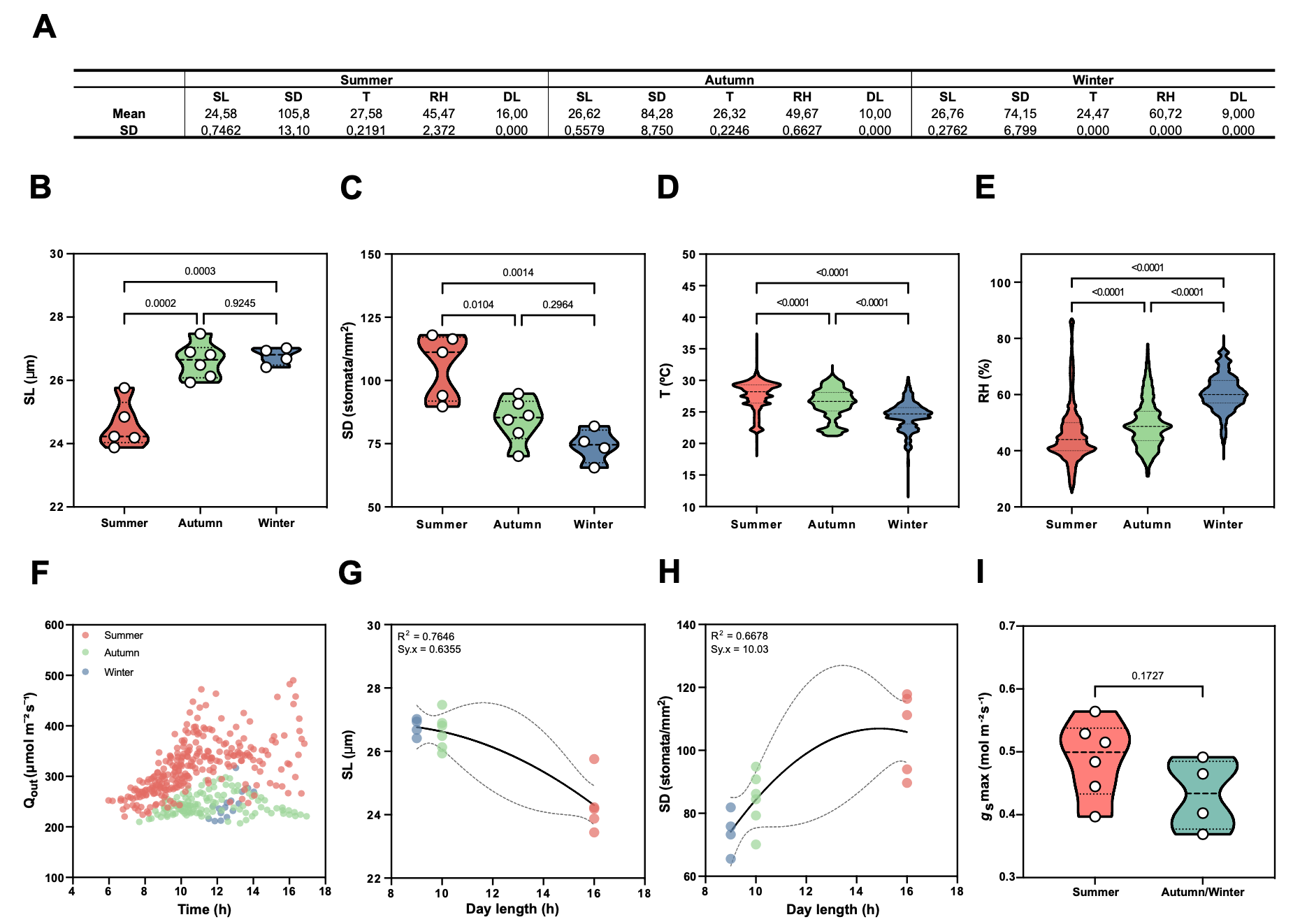
**

**Figure S3 - Seasonal effects on stomatal anatomy and environmental conditions.** (A) Mean values and standard deviation of stomatal length (SL), stomatal density (SD), average growth temperature (T), average growth relative humidity (RH) and day length (DL) for the summer, autumn and winter plants. (B) SL seasonal variation (summer n=5, autumn n=6, winter n=4). (C) SD seasonal variation (summer n=5, autumn n=6, winter n=4).). (D) Growth temperature variation for each season 2 weeks before leaf collection. (E) Growth relative humidity variation for each season 2 weeks before leaf collection. (F) Daily variation in ambient light intensity (data from LI-6800 measurements). (G) Non-linear relation between SL and day length. (H) Non-linear relation between SD and day length. (I) Physiological maximum stomatal conductance (*g*_s_max) of summer (n=6) and autumn/winter (n=3/n=1, respectively) plants. The *g*_s_max measurements were not performed on the plants that were anatomically assessed, which is why the number of individuals varies (summer n=6, fall n=3, winter n=1) and why autumn and winter measurements were combined. R^2^ and Sy.x or p-values are indicated.

**
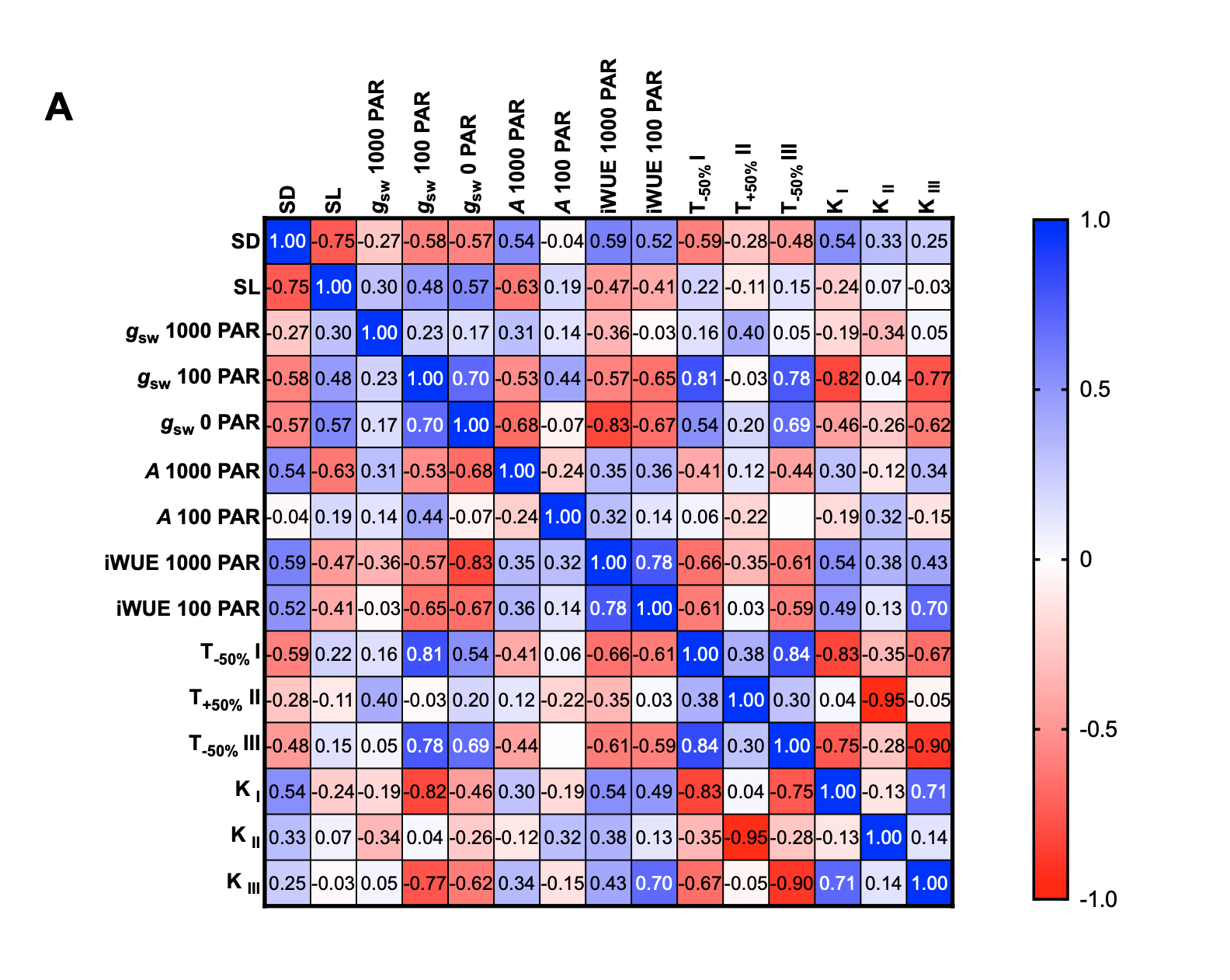
**

**Figure S4 - Anatomical influence on gas exchange and stomatal kinetics.** (A) Correlation matrix between anatomical traits (SD and SL), steady-state *g*_sw_ (at 1000, 100 and 0 PAR), steady-state *A* (at 100 and 1000 PAR), iWUE (1000 and 100 PAR), half-time (for the light transitions 1000-100 (T_-50%_ I), 100-1000 (T_+50%_II) and 1000-0 PAR (T_-50%_III)) and constant rate (for the light transitions 1000-100 (K _I_), 100-1000 (K_II_) and 1000-0 PAR (K_III_)); n=15.

**
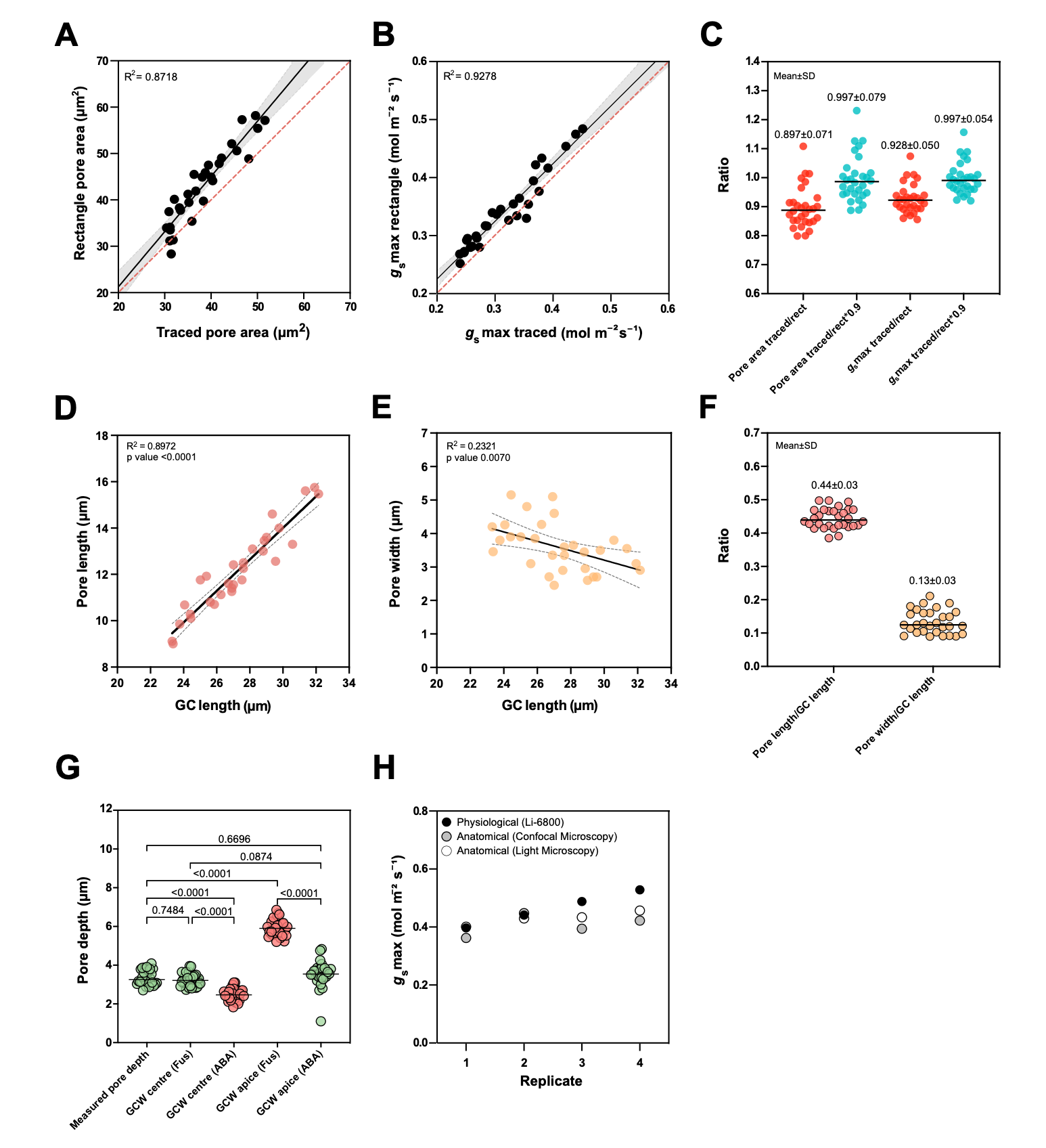
**

**Figure S5 - Quantitative morphometry of the graminoid stomata in *B. distachyon* Bd21-3.** (A) Linear regression between hand-traced pore area and rectangle pore area. (B) Linear regression of anatomical *g*_s_max calculated with hand-traced pore area and with rectangle pore. (C) Ratios between hand-traced and rectangle pore, hand-traced and rectangle*0.9, anatomical gsmax calculated with hand-traced and rectangle pores, and anatomical gsmax calculated with hand-traced and rectangle*0.9 pores. (D) Linear regression between guard cell length (GCL) and pore length (PL). (E) Linear regression between guard cell length (GCL) and pore width (PW). (F) Ratios between GCL and PL and between GCL and PW. (G) Comparison of measured pore depth (l) with itself, GCW_C_ (fusicoccin-treatment), GCW_C_ (ABA-treatment), GCW_A_ (fusicoccin-treatment) and GCW_A_ (ABA-treatment). Green groups indicate that the measured parameters were a good indication of pore depth (l), red groups were not. (H) Physiological (IRGA-based), anatomical (light microscopy-based) and anatomical (confocal-based) *g*_s_max values of each replicate shown in Fig. 5I. R^2^ and p-values are indicated.

**Supplementary Dataset 1 - Gas exchange and anatomical data measured and analyzed in this study.**
