# Supplementary figures and images for: Quantitative effects of environmental variation on stomatal anatomy and gas exchange in a grass model
Source: Quant Plant Biol. 2022 Mar 9;3:e6. doi: 10.1017/qpb.2021.19 (PMC10095872; doi:10.1017/qpb.2021.19)

**A**

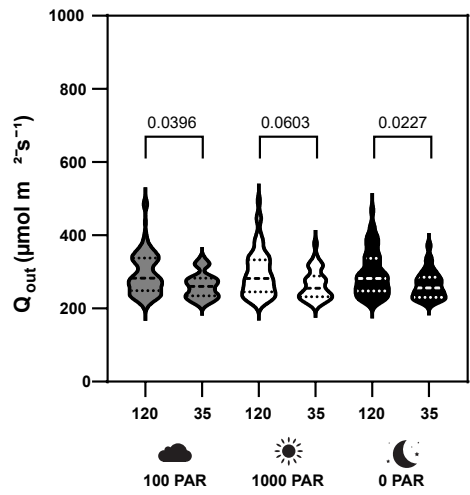

**B**

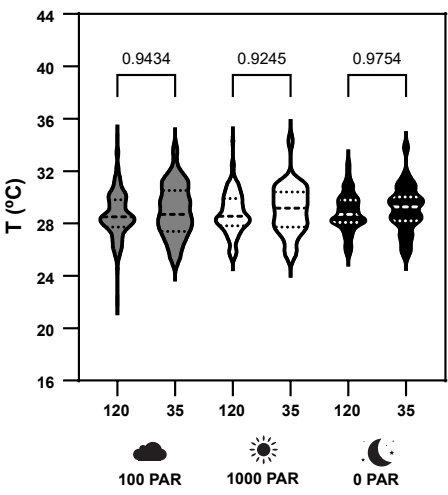

**C**

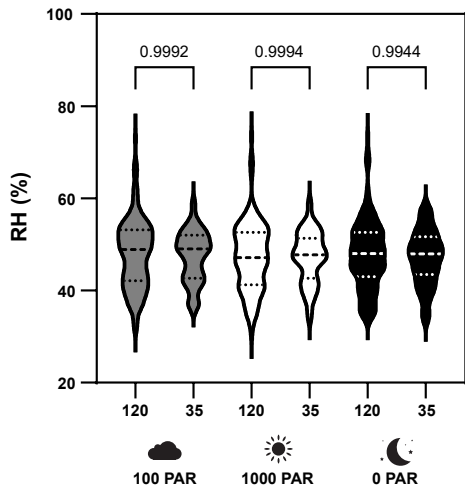

**D**

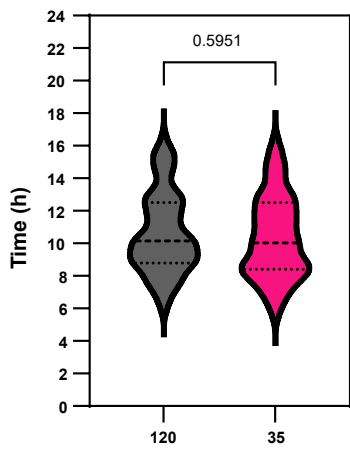

**E**

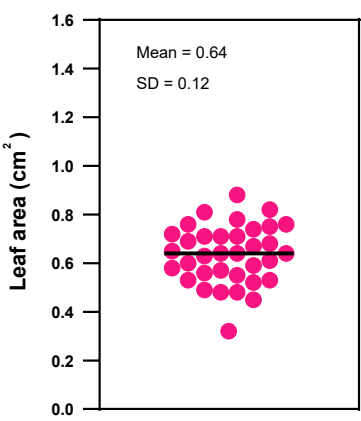

**F**

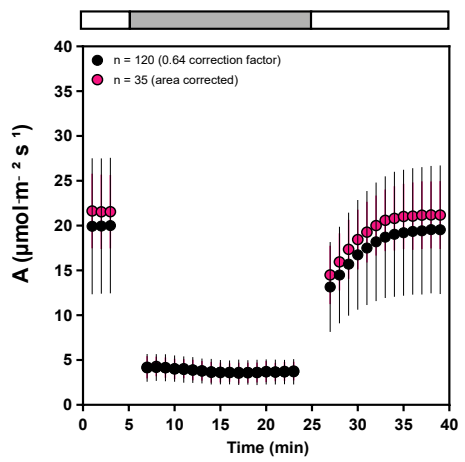

Supplement: Supplementary file 1 [file S2632882821000199sup001.zip › S2632882821000199sup003.pdf]

A

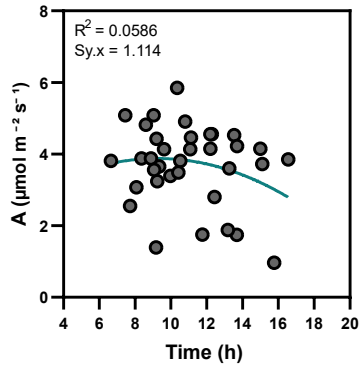

B

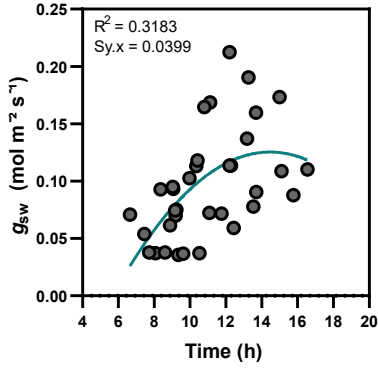

C

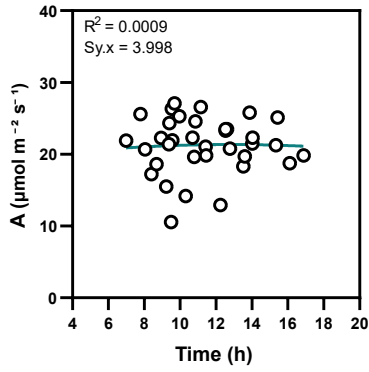

D

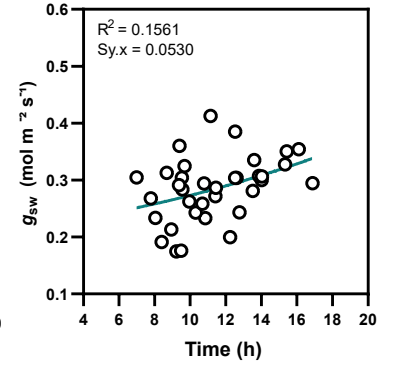

E

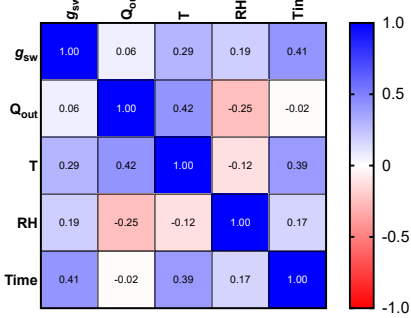

F

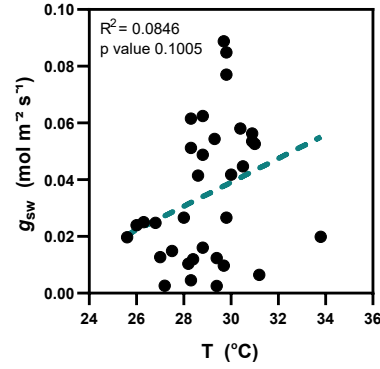

G

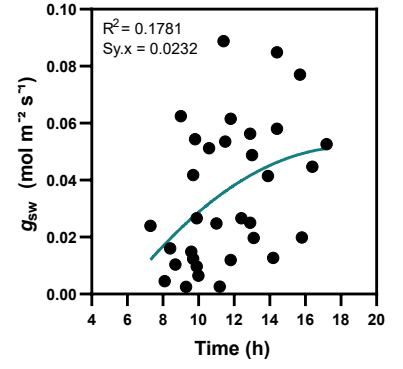

H

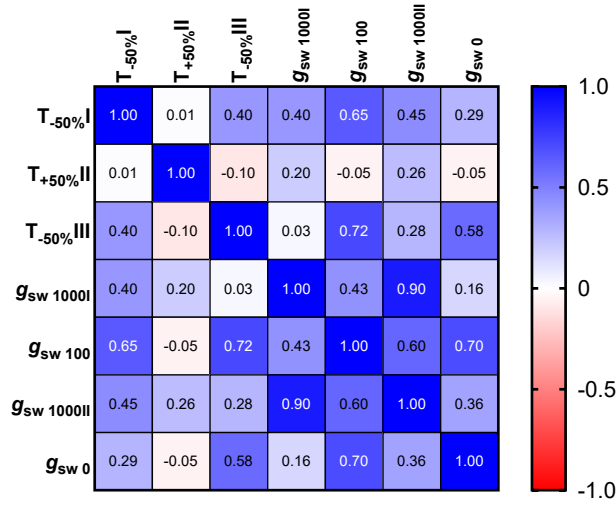

I

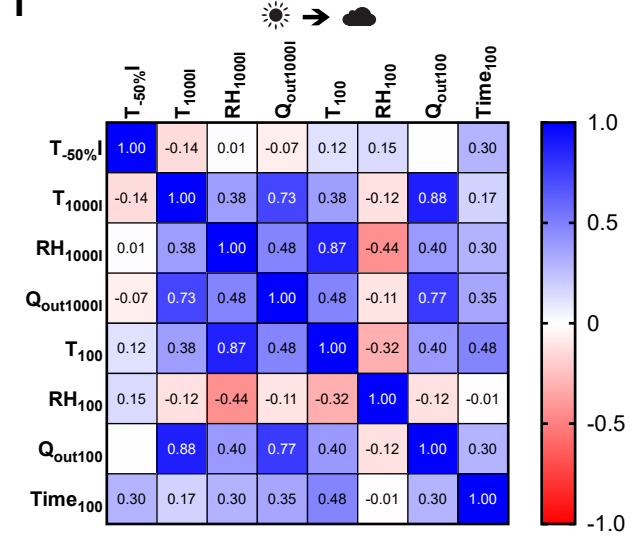

J

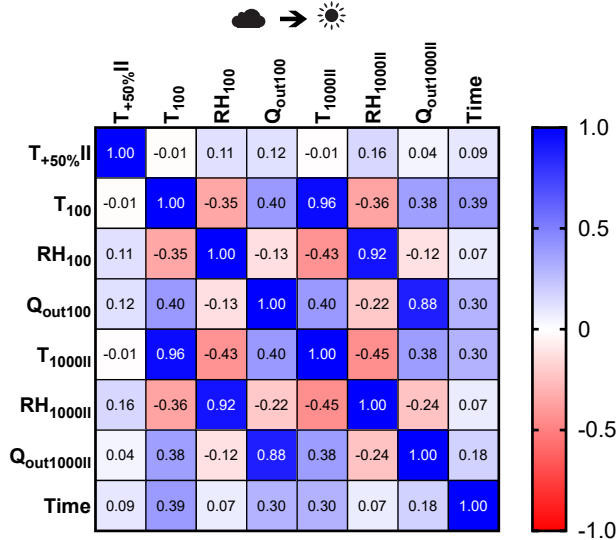

K

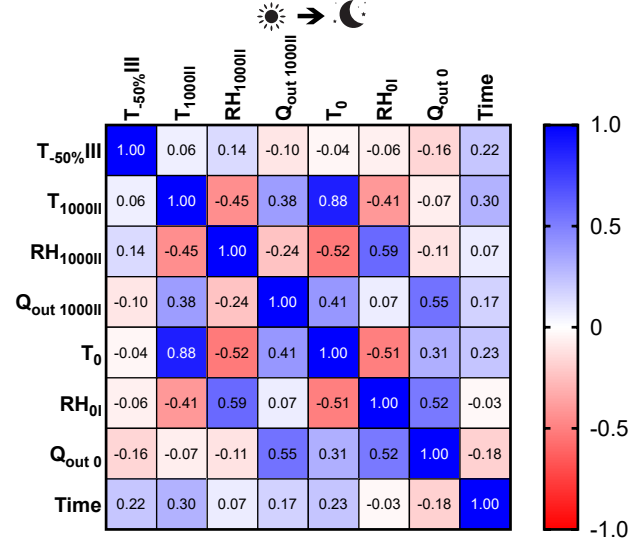

Supplement: Supplementary file 1 [file S2632882821000199sup001.zip › S2632882821000199sup004.pdf]

A

|      | Summer |       |        |       |       | Autumn |       |        |        |       | Winter |       |       |       |       |
|------|--------|-------|--------|-------|-------|--------|-------|--------|--------|-------|--------|-------|-------|-------|-------|
|      | SL     | SD    | T      | RH    | DL    | SL     | SD    | T      | RH     | DL    | SL     | SD    | T     | RH    | DL    |
| Mean | 24,58  | 105,8 | 27,58  | 45,47 | 16,00 | 26,62  | 84,28 | 26,32  | 49,67  | 10,00 | 26,76  | 74,15 | 24,47 | 60,72 | 9,000 |
| SD   | 0,7462 | 13,10 | 0,2191 | 2,372 | 0,000 | 0,5579 | 8,750 | 0,2246 | 0,6627 | 0,000 | 0,2762 | 6,799 | 0,000 | 0,000 | 0,000 |

B

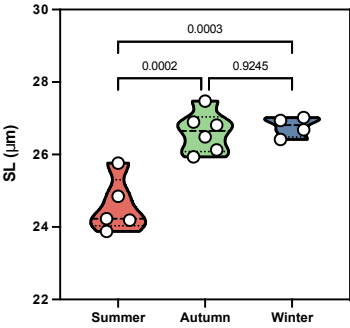

C

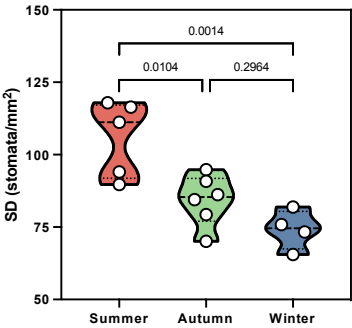

D

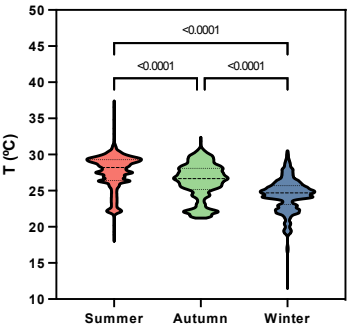

E

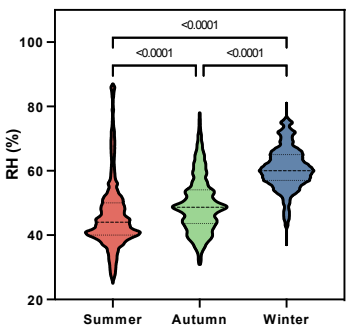

F

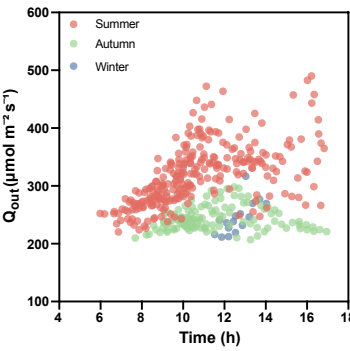

G

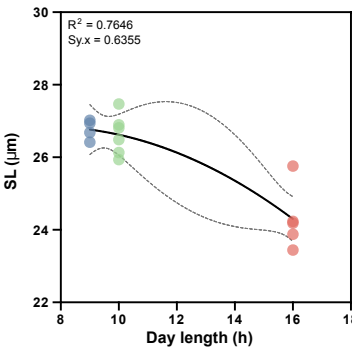

H

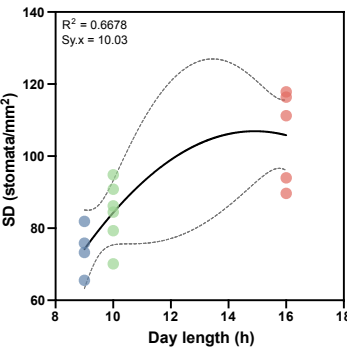

I

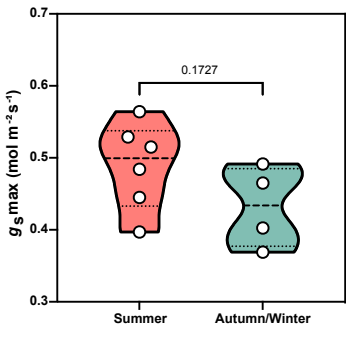

Supplement: Supplementary file 1 [file S2632882821000199sup001.zip › S2632882821000199sup005.pdf]

A

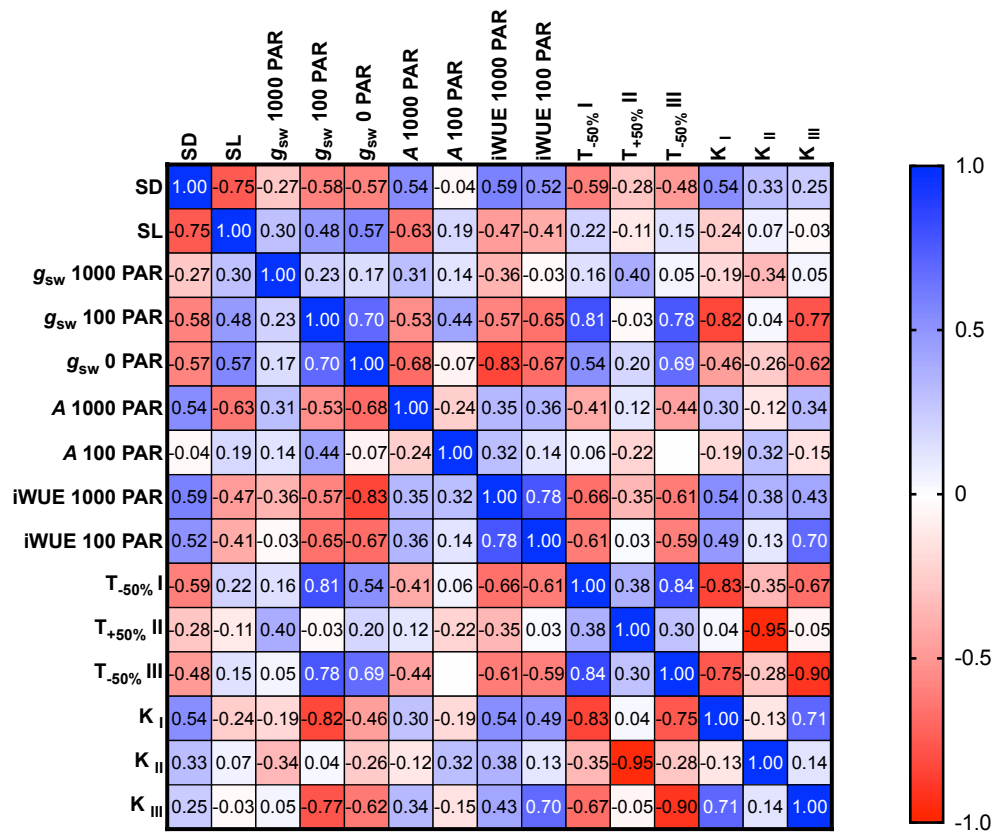

Supplement: Supplementary file 1 [file S2632882821000199sup001.zip › S2632882821000199sup006.pdf]

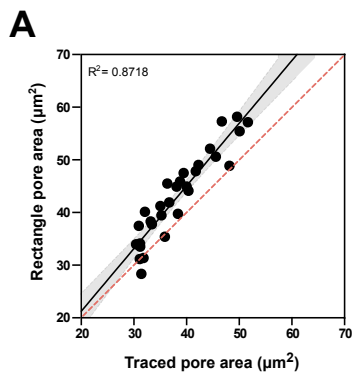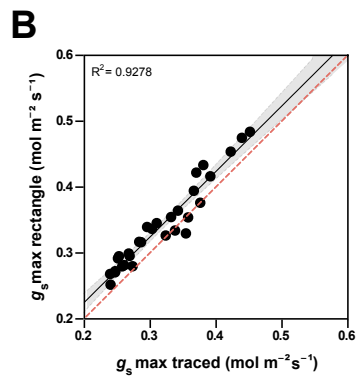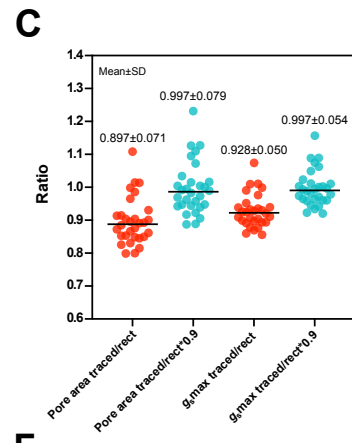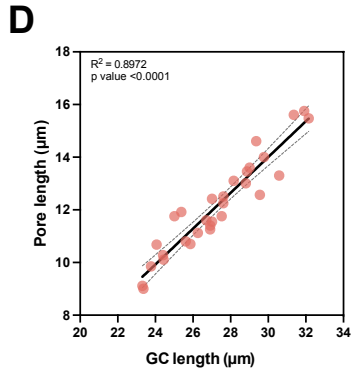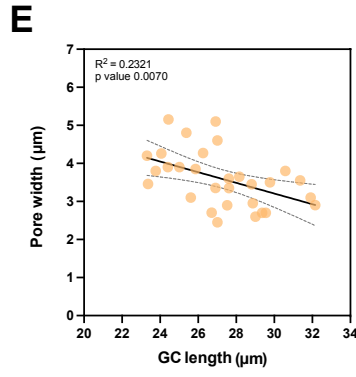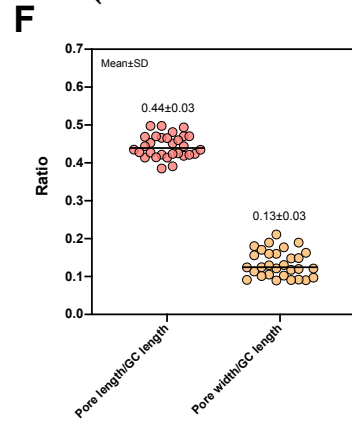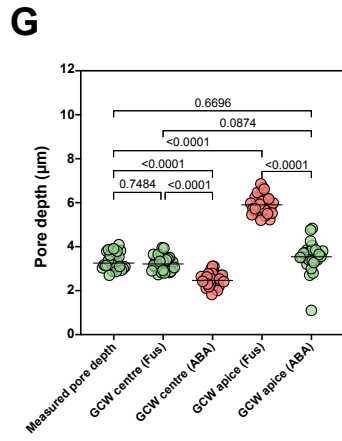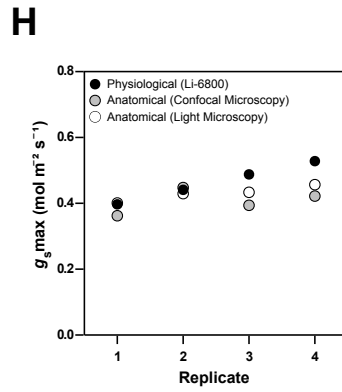

Supplement: Supplementary file 1 [file S2632882821000199sup001.zip › S2632882821000199sup007.pdf]
